# Assessment of the current status of real-world pharmacogenomic testing: informed consent, patient education, and related practices

**DOI:** 10.3389/fphar.2024.1355412

**Published:** 2024-02-08

**Authors:** Lucas Pereira, Cyrine-Eliana Haidar, Susanne B. Haga, Anna G. Cisler, April Hall, Sanjay K. Shukla, Scott J. Hebbring, Emili J. W. Leary

**Affiliations:** ^1^ Marshfield Clinic Research Institute, Center for Precision Medicine Research, Marshfield Clinic Health Systems, Marshfield, WI, United States; ^2^ Division of Genetics and Metabolism, Department of Pediatrics, School of Medicine and Public Health, University of Wisconsin-Madison, Madison, WI, United States; ^3^ Department of Pharmacy and Pharmaceutical Sciences, St. Jude Children’s Research Hospital, Memphis, TN, United States; ^4^ Program in Precision Medicine, Department of Medicine, School of Medicine, Duke University, Durham, NC, United States; ^5^ Medical Genetics, Marshfield Clinic Health Systems, Marshfield, WI, United States; ^6^ Center for Human Genomics and Precision Medicine, Wisconsin Institute for Medical Research, University of Wisconsin-Madison, Madison, WI, United States

**Keywords:** clinical implementation, genetic counseling, genetic testing, informed consent, pharmacogenetics, pharmacogenomics

## Abstract

**Introduction:** The practice of informed consent (IC) for pharmacogenomic testing in clinical settings varies, and there is currently no consensus on which elements of IC to provide to patients. This study aims to assess current IC practices for pharmacogenomic testing.

**Methods:** An online survey was developed and sent to health providers at institutions that offer clinical germline pharmacogenomic testing to assess current IC practices.

**Results:** Forty-six completed surveys representing 43 clinical institutions offering pharmacogenomic testing were received. Thirty-two (74%) respondents obtain IC from patients with variability in elements incorporated. Results revealed that twenty-nine (67%) institutions discuss the benefits, description, and purpose of pharmacogenomic testing with patients. Less commonly discussed elements included methodology and accuracy of testing, and laboratory storage of samples.

**Discussion:** IC practices varied widely among survey respondents. Most respondents desire the establishment of consensus IC recommendations from a trusted pharmacogenomics organization to help address these disparities.

## 1 Introduction

Precision medicine is becoming increasingly integrated into standard healthcare practice (Carrasco-Ramiro et al., 2017; Goetz and Schork, 2018). Pharmacogenomic testing, which analyzes genetic variation associated with drug metabolism and/or response in order to tailor pharmacotherapy (Wake et al., 2019), is one of the key applications of precision medicine. Pharmacogenomic testing can be ordered either in a reactive (when medication use is being considered or after medication initiation and an adverse response or no response experienced) or pre-emptive manner (in advance of medication needs) (Haidar et al., 2022).

The American Medical Association defines informed consent (IC) as the process of communication that occurs between a patient and a healthcare provider, which results in the patient’s authorization or agreement to undergo a specific medical intervention (AMA, 2013), such as genetic testing. This is typically obtained for single-gene, chromosomal disorder testing, as well as whole exome or genome sequencing (WES or WGS, respectively), to ensure patient understanding of testing expectations and allow for a more thorough discussion of questions or concerns the patient may have before electing to proceed (Rieger and Pentz, 1999; Rego et al., 2019). Pre-test genetic counseling is generally done to provide the patient an opportunity to discuss such implications of genetic testing with a genetics specialist (such as a medical geneticist, genetic counselor (GC), or pharmacist in the case of pharmacogenomic testing) and as a means of obtaining IC. As patient education is a core component of IC, it is challenging to deliver a wide array of complex information about testing in a manner that will optimize patient understanding (Perrenoud et al., 2015). Given this complexity and the limited time providers have, there is wide variation in IC practices across medical specialties (Glaser et al., 2020; Kaebnick, 2021; Singer et al., 2022).

Elements of IC that are typically discussed during pre-test genetic counseling for disease-based testing generally fall into eight categories: a general description of the test, the purpose of the test, whether genetic counseling is recommended, possible results and implications, a description of the condition being tested, disclosure of the results, storage/destruction of the biological sample, and medical risks and benefits associated with undergoing the test (Committee on Energy and Commerce, 2008; Haga and Mills, 2016). Some or all of these elements are required by certain state laws (Spector-Bagdady et al., 2018), such as New York state which has more stringent laws regarding genetic testing (New York Consolidated Laws, Civil Rights Law - CVR §79-L, 2021). Recommendations regarding IC practices for genetic conditions, such as hereditary cancer syndromes (e.g., hereditary breast and ovarian cancer, hereditary non-polyposis colon cancer, etc.), Huntington disease, and WES/WGS have been developed (Lucassen and Hall, 2019; Yu et al., 2019) but are lacking for pharmacogenomic testing.

Pharmacogenomic testing is typically perceived as having low overall risks compared to other types of clinical genetic testing due to a number of reasons (Payne et al., 2011; Zhang et al., 2014). Whereas disease-based genetic testing may predict risk for future health concerns and have increased risk for familial implications, pharmacogenomic testing only queries genes with a medication response implication, thus health and relatedness findings are less likely. While it is theoretically possible to discriminate based upon medication response, the lack of health implications may reduce discrimination concerns. Regardless, the Genetic Information Nondiscrimination Act of 2008 (GINA) protects patients from discrimination by health insurance companies and employers of more than 15 employees on the basis of genetic test results (Committee on Energy and Commerce, 2008). Thus, pharmacogenomic testing may avoid some of the ethical and insurability issues compared to disease-based genetic testing. This is suggested by patients often not perceiving risks to employment or health insurance with pharmacogenomic testing (McCarthy et al., 2020; Stancil et al., 2021). It is possible, however, for pharmacogenomic tests to reveal information regarding disease risk (known as incidental or secondary findings) since pharmacogenes may also be involved in disease-related pathways in addition to their role in drug metabolism or transport, such as Gilbert syndrome (Strassburg, 2008) or Factor V Leiden (Zhang et al., 2018).

The setting in which pharmacogenomic testing is ordered may also influence the IC and delivery process. To date, pharmacogenomic testing has often been conducted in the research setting in implementation studies; clinical pharmacogenomic testing has only recently gained traction in routine medical practice (Duarte and Cavallari, 2021). The research setting requires full IC from the research participants in compliance with human subjects regulations (Howard et al., 2011; Moran et al., 2011; Shaw et al., 2013). In the clinical setting, however, no explicit guidance currently exists about what or how much education and/or IC is required before pharmacogenomic testing.

In 2016, Haga and Mills reviewed clinical pharmacogenomic testing laboratories and found a range of variability in the extent to which IC is required. It is important to note this study did not delve beyond the laboratory practices into the clinical side of IC practices. Clinical laboratories may require provider attestation or patient signature regarding IC on the pharmacogenomic test requisition form, but do not specify what information should be disclosed to patients (Pamarti, 2011).

There is currently no consensus on how providers should approach the discussion and possible delivery of incidental findings with patients (Brothers et al., 2013; Haga, 2021), though there is evidence supporting clinicians’ desire to provide this and other elements of IC to patients in the setting of pharmacogenomic testing (Muflih et al., 2020). While it is widely recognized that there is a need for guidance and standardization of IC for clinical pharmacogenomic testing (Avard et al., 2009; Haga and Mills, 2016; Ando and Terada, 2023), the IC practices of clinical providers ordering pharmacogenomic testing has not been reported in the literature to date. In order to improve and establish standardized IC practices within the pharmacogenomics community, the first step is to bridge this gap in knowledge by determining the current status of IC practices and assess opinions of key stakeholders within the community. In this study, we report the findings from a multi-institutional survey on current IC practices for clinical pharmacogenomic testing.

## 2 Methods

### 2.1 Study design and participants

To collect information on IC practices from institutions that have implemented pharmacogenomic testing, we developed a REDCap-based questionnaire (Supplementary Appendix SA). Questions regarding elements of IC were modeled from the Haga and Mills study of clinical laboratory pharmacogenomic testing (2016). The REDCap survey length was variable as skip logic and branching were employed, with up to 45 questions pertaining to test utilization and IC/assent for adult and/or pediatric patients and elements of IC incorporated in pre-test counseling (e.g., benefits, risks, utility, incidental findings, etc.). Other questions asked about laboratory selection, testing methodology, and whether testing for pharmacogenes with incidental findings or any other factors impacted their IC process. The question format was a combination of multiple choice or open answer. All questions were programmed to be optional to improve the response rate. Questions were also asked to analyze demographics of provider respondents. Surveys with incomplete demographic information were included in the dataset if the implementation questions were completed. Respondents had the option to submit contact information for follow-up in case further clarification was necessary.

The survey invitation was sent to listserv email blasts to over 5000 individuals consisting of members of the Clinical Pharmacogenetics Implementation Consortium (CPIC), National Society of Genetic Counselors (NSGC), and the pharmacogenetics working groups of the Electronic Medical Records and Genomics (eMERGE) and the Inter-Society Coordinating Committee for Practitioner Education in Genomics (ISCC-PEG). We selected these organizations in order to target individuals who would represent the pharmacogenomics expert and/or be able to speak knowledgeably about the pharmacogenomics IC practices at their respective clinical practice site. The survey was conducted during February and March of 2023. Regardless of whether pharmacogenomic testing was offered at their institution, all respondents who took the survey were asked what resources would be helpful in further enhancement of clinical pharmacogenomics implementation services.

### 2.2 Data analysis

Descriptive statistics were generated for responses to each survey question. In the instance of multiple respondents from one institution, we deferred to the answers provided by the provider denoted to be directly involved in the IC process at their institution in order to better reflect their actual practices. When discrepancies could not be resolved in this manner, we elected to follow majority rule for data representation of IC practices.

### 2.3 Ethics approval

Institutional Review Board exemption was obtained from all institutions with which the investigators actively involved in disseminating the survey are affiliated (Marshfield Clinic Research Institute IRB-22-1108; University of Wisconsin-Madison IRB-2022-1632; St. Jude Children’s Research Hospital IRB-23-1274).

## 3 Results

### 3.1 Demographics

**FIGURE 1:**
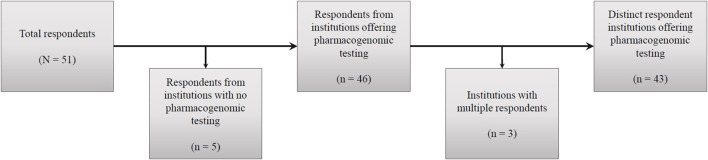
Survey responses by institution.

**FIGURE 2:**
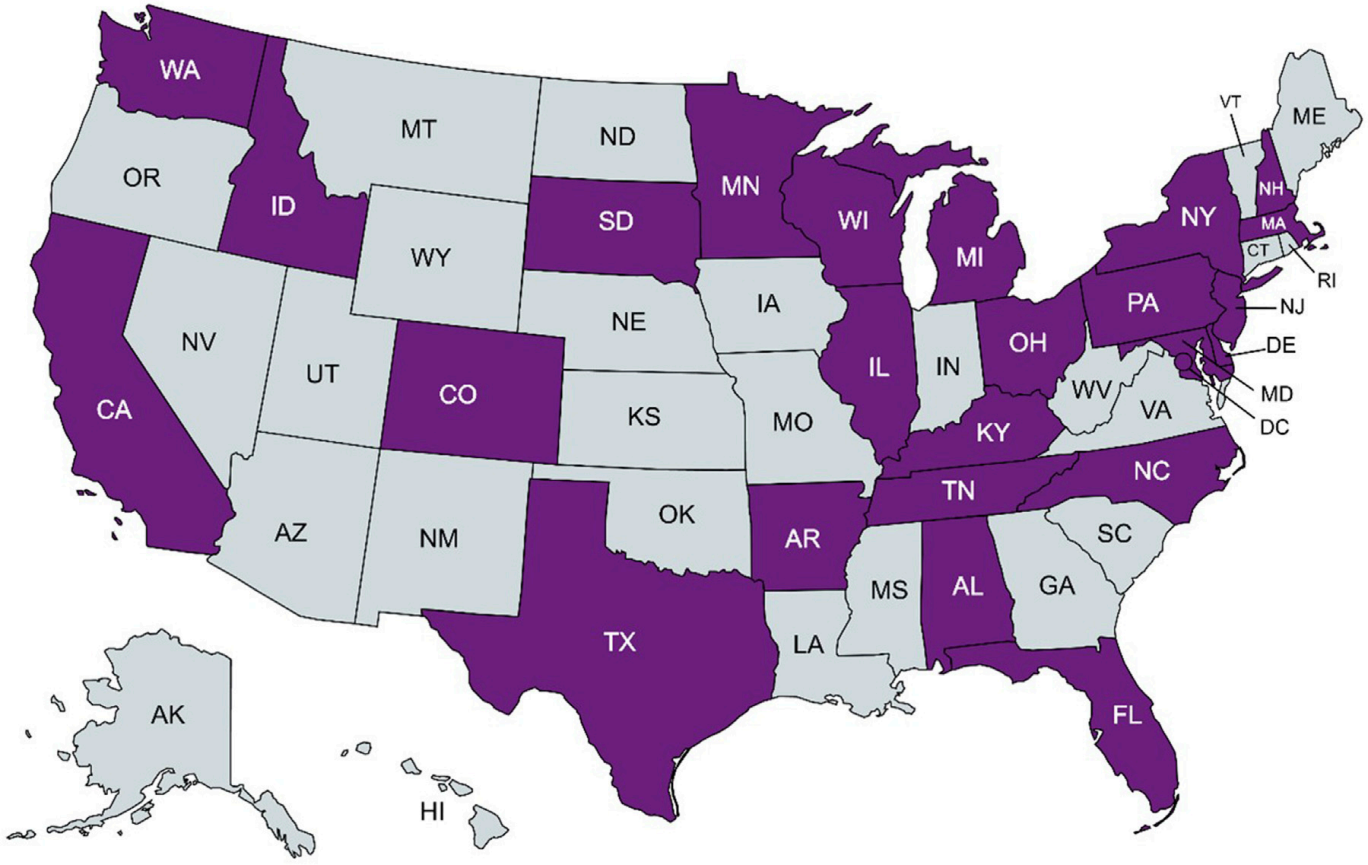
States within the United States with a clinical pharmacogenomic respondent institution in purple. Created with MapChart.

A total of 51 respondents completed the survey, representing 48 institutions. These included a variety of clinical settings, including academic medical centers, research hospitals, private practices, and public hospitals. Responses from institutions (n = 5) that do not order pharmacogenomic testing were omitted, resulting in a total of 46 responses from 43 distinct institutions with pharmacogenomics implementation (Figure 1), representing 24 states within the U.S. and the District of Columbia (Figure 2), as well as Alberta, Canada; Jalisco, Mexico; Salzburg, Austria, and Singapore. Two institutions did not provide a location; however, their overall responses were distinct from all others and were counted as independent institutions.

Of these 43 institutions, 15 (35%) institutions offered clinical pharmacogenomic testing only to adult patients and four (9%) only to pediatric patients, with 24 (56%) offering testing to both populations (Table 1). Out of the 39 institutions that offered testing to adults, 15 (39%) respondents indicated that they ordered 1–10 pharmacogenomic tests per month, two (5%) ordered 11-20 tests per month, and 16 (41%) have ordered more than 20 tests per month over the last year. Six respondents (15%) indicated that they did not know how many tests were ordered at their institution or did not order any pharmacogenomic testing for adult patients. Out of the 28 institutions that offered testing to pediatric patients, 11 (39%) indicated that they ordered 1–10 pharmacogenomic tests per month, one (4%) ordered 11-20 tests per month, and six (21%) have ordered more than 20 tests per month over the last year. Ten respondents (representing 36% of surveyed institutions) indicated that they did not know how many tests were ordered or did not order any pharmacogenomic testing for pediatric patients. A vast majority (93%) of institutions offered testing in an outpatient (ambulatory) clinic setting. Just over half (54%) of these institutions also offered pharmacogenomic testing in an inpatient (hospital) setting. Six (14%) institutions offered pharmacogenomic testing to patients directly through an industry (i.e., commercial laboratory with patient-facing services) setting; of note, only two (5%) institutions offered this as the *only* setting for testing (Table 1).

**TABLE 1:** Institution demographics (*n* = 43).

	USA based *n* = 38	Outside USA *n* = 5	All respondents *n* = 43
Patient population tested			
Adults Only	13 (34%)	2 (5%)	15 (35%)
Pediatrics Only	4 (10%)	0 (0%)	4 (9%)
Adults & Pediatrics	21 (55%)	3 (7%)	24 (56%)
Clinical Setting			
Outpatient Only	17 (45%)	0 (0%)	17 (40%)
Outpatient & Inpatient	17 (45%)	2 (40%)	19 (44%)
Industry Only	1 (2%)	1 (20%)	2 (5%)
All of the above	3 (8%)	1 (20%)	4 (9%)
Not indicated	0 (0%)	1 (20%)	1 (2%)

Most of the 46 respondents (65%) were pharmacists. The demographics of pharmacists and non-pharmacist respondents are summarized in Tables 2, 3 respectively. Just over half (54%) of all respondents have been practicing in their field for at least 6 years. The type of training respondents received in pharmacogenomics ranged from on-the-job training (50%) to a Master’s degree in pharmacogenomics (4%). Most respondents (65%) reported only receiving pharmacogenomics training outside of their terminal degree education. A majority (60%) of pharmacist respondents did not receive pharmacogenomics training in pharmacy school. All GCs surveyed received pharmacogenomics training through on-the-job training, with just over half (55%) having also pursued continuing education. Four of the respondents were medical doctors (9%), three of whom have pursued continuing education in pharmacogenomics, with two having completed a pharmacogenomics certificate course. The three remaining respondents were PhD pharmacogenomics implementers (7%) each with varying types of pharmacogenomics training, including on-the-job training, continuing education, and a specialized PhD in pharmacogenomics.

**TABLE 2:** Pharmacist pharmacogenomics training by years of experience (*n* = 30).

	<1 year experience	1–2 years experience	3–5 years experience	6–10 years experience	>10 years experience	n^a^
*n*	3	3	8	7	9	30
Pharmacogenomics certificate course	0	2	3	3	5	13
Formal education	2	1	2	2	5	12
PGY2 in Pharmacogenomics	2	1	4	3	1	11
Continuing education	1	2	1	3	4	11
On the job training	1	0	0	3	5	9
Pharmacogenomics fellowship	1	1	5	0	0	7
Master’s degree in Pharmacogenomics	0	0	0	1	1	2

^a^ Respondents may have acquired multiple forms of pharmacogenomics training.

**TABLE 3:** Demographics for non-pharmacist pharmacogenomics professionals (*n* = 16).

	<1 year experience	1–2 years experience	3–5 years experience	6–10 years experience	>10 years experience	n^a^
**Genetic Counselor**	**0**	**1**	**3**	**2**	**3**	**9**
Pharmacogenomics certificate course	0	0	0	0	0	0
Formal education	0	0	2	1	0	3
Continuing education	0	0	2	1	2	5
On the job training	0	1	3	2	3	9
**Medical Doctors**	**1**	**0**	**1**	**0**	**2**	**4**
Pharmacogenomics certificate course	1	0	1	0	0	2
Formal education	0	0	0	0	0	0
Continuing education	1	0	1	0	1	3
On the job training	1	0	1	0	2	4
**PhD Implementers**	**0**	**0**	**1**	**1**	**1**	**3**
Pharmacogenomics certificate course	0	0	0	0	0	0
Formal education	0	0	1	0	0	1
Continuing education	0	0	0	0	1	1
On the job training	0	0	0	1	0	1

^a^ Respondents may have acquired multiple forms of pharmacogenomics training.

### 3.2 Informed consent practices

**FIGURE 3:**
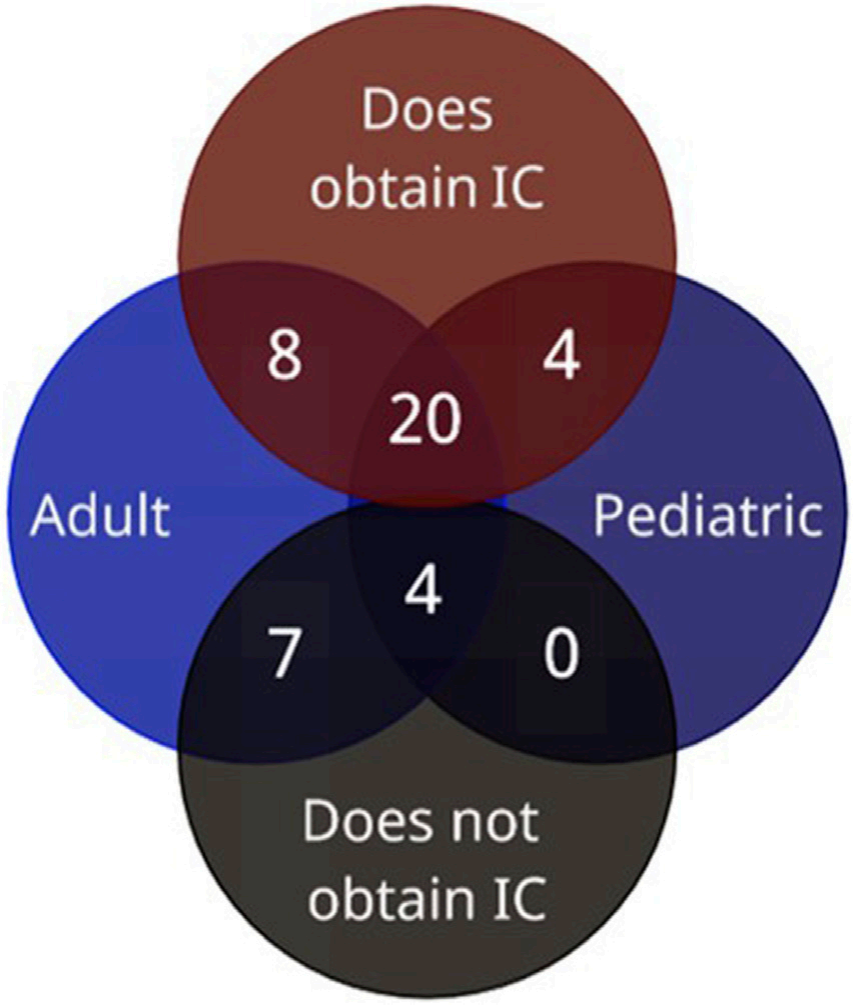
Institutions’ IC practices by age demographic (*n* = 43).

Thirty-two of the 43 (74%) institutions obtained IC prior to clinical pharmacogenomic testing. One institution (2%) reported offering IC for both adults and pediatrics; however, they reportedly did not know the details of what was offered to either; excluding this respondent from the dataset resulted in 31 institutions with evaluable IC data. Of note, there were three (7%) institutions that provided IC to both adults and pediatric patients; however, they indicated they did not know the exact elements incorporated for pediatric patients. Of the 11 (26%) that do not obtain IC, seven (16%) offered testing only to adults, and four (9%) offered testing to both adult and pediatric patients (Figure 3).

Of the 31 institutions that obtained and reported specific IC practices, 26 reported their IC practices for adult patients and 20 for parents of pediatric patients (Table 4). All four institutions that only ordered pharmacogenomic testing for pediatric patients provided IC to parents (Figure 3). Elements of IC for pharmacogenomic testing most commonly included across institutions were 1) a general description of the test (*n* = 29; 93%), 2) purpose of the test (*n* = 28; 90%), and 3) the potential benefit of assisting with medication selection/avoidance (*n* = 27; 87%). Elements least commonly included in IC were 1) discussion of laboratory storage and usage of the sample (*n* = 14; 45%), 2) methodology limitations to testing (*n* = 14; 45%), and 3) accuracy of testing (*n* = 15; 48%) (Table 4). While the survey responses indicated current pharmacogenomic testing offerings included most of the same elements as associated with disease-based genetic testing, there was a key difference of the “description of the condition being tested” (Table 5). The specimen type used for testing was not queried in the survey; as such, that data is not available. The two institutions with multiple respondents had a concordance rate of 70%–75% between responses regarding IC practices.

**TABLE 4:** Components of informed consent utilized in clinical pharmacogenomics testing (*n* = 31).

Component of consent	Institutions offering informed consent in adults *n* = 26 (%) (n_Adult_ = 7; n_Both_ = 19)	Institutions offering informed consent in pediatrics *n* = 20 (%) (n_Pediatric_ = 4; n_Both_ = 16)	Institutions offering informed consent in both adults and pediatrics *n* = 16 (%)	All institutions offering informed consent *n* = 31 (%)
Benefits of testing				
Assist with medication selection and dosing	23 (88)	18 (90)	14 (88)	27 (87)
Lifetime use of results	20 (77)	17 (85)	13 (81)	24 (77)
Risks/considerations of testing				
Patient protections (e.g., GINA, HIPAA, State Laws, etc.)	19 (73)	16 (80)	12 (75)	23 (74)
Description of incidental findings	19 (73)	15 (75)	12 (75)	22 (71)
Implications of results for biologically related individuals	12 (46)	14 (70)	10 (63)	16 (52)
When genetic counseling may be recommended	14 (54)	13 (65)	11 (69)	16 (52)
Storage and use of sample for validation, research, etc.	12 (46)	10 (50)	8 (50)	14 (45)
Test education/use of results				
General description of the test	25 (96)	19 (95)	15 (94)	29 (93)
Purpose of testing	24 (92)	19 (95)	15 (94)	28 (90)
Accuracy of testing	13 (50)	12 (60)	10 (63)	15 (48)
Actionability of results	20 (77)	16 (80)	11 (69)	24 (77)
General limitations to testing	21 (81)	16 (80)	12 (75)	24 (77)
Methodology limitations to testing	13 (50)	11 (55)	9 (56)	14 (45)
Cost of testing	22 (85)	16 (80)	12 (75)	25 (81)
Interpretation of results	21 (81)	18 (90)	12 (75)	26 (84)

**TABLE 5:** Comparison of the eight elements of informed consent of disease-based genetic testing to pharmacogenomic testing survey participant responses (*n* = 31).

Element included in IC (*and survey examples as appropriate*)	Adult IC n = 26 (%) (n_Adult_ = 7; n_Both_ = 19)	Pediatric IC n = 20 (%) (n_Pediatric_ = 4; n_Both_ = 16)	Both adult and pediatric IC n = 16 (%) median %
General description of the test	25 (96)	19 (95)	15 (94)
Purpose of the test	24 (92)	19 (95)	15 (94)
Whether genetic counseling is recommended	14 (54)	13 (65)	11 (69)
Possible results and implications			
Description of incidental findings	19 (73)	15 (75)	12 (75)
Implications of results for biologically related individuals	12 (46)	14 (70)	10 (63)
Description of the condition being tested	N/A	N/A	N/A
Disclosure of the results			
Patient protections (e.g., GINA, HIPAA, State Laws, etc.)	19 (73)	16 (80)	12 (75)
Storage/destruction of the biological sample	12 (46)	10 (50)	8 (50)
Medical risks and benefits associated with undergoing the test			
Assist with medication selection and dosing	23 (89)	18 (90)	14 (88)
Lifetime use of results	20 (77)	17 (85)	13 (81)

N/A, not applicable.

All but two institutions that obtained IC for adult and pediatric patients reported using the same elements of IC for both patient populations. In some cases, additional information was provided to parents of pediatric patients; one institution discussed implications for biologically related relatives and laboratory storage and usage of the sample for pediatric patients, but not for adult patients. Another institution discussed actionability and interpretation of results with parents of pediatric patients, but not with adult patients, and discussed the cost of testing with adult patients but not with parents of pediatric patients. The greatest disparity in elements of IC between the two populations observed in our data is discussion of the implications of pharmacogenomic test results for biologically related individuals, with a 24% difference between the adult and pediatric patient populations; however, despite being the largest disparity, it does not meet the threshold for statistical significance (*p* = 0.067). Most (84%) of the institutions that obtained IC reported using multiple methods, such as in-person, telehealth, and/or written communication (e.g., electronically signed consent form). Of the 15 institutions that obtained IC for both adult and parents of pediatric patients, only one reported using different methods for each population (i.e., only obtaining IC for parents of pediatric patients in-person or over telehealth video but using a different method for adult patients).

Respondents identified that various healthcare providers are involved with pre-test education, obtaining IC, and ordering clinical pharmacogenomic testing including pharmacists (70% of institutions offering adult pharmacogenomic testing and 75% offering pediatric pharmacogenomic testing) and/or GCs (37% of institutions offering adult pharmacogenomics and 25% offering pediatric pharmacogenomics). Eight respondents indicated that pre-test counseling is a collaborative effort between multiple healthcare providers for pediatric and/or adult patients, often being pharmacists and GCs (*n* = 5), although other providers may also be involved (Table 6). All GC respondents worked at an institution that obtained IC from patients.

**TABLE 6:** Obtainment of IC for pharmacogenomic testing (*n* = 31).

	Adult *n* = 26	Pediatric *n* = 20
Most frequent provider to obtain IC^a^		
Pharmacist	19	15
Genetic counselor	10	5
Physician	6	4
Nurse	3	3
Method of Obtaining IC^b^		
In person	18	16
By phone	15	10
By telehealth video	14	12
Written communication	10	8
Documentation of IC		
Signed consent stored in health record	8	7
Verbal consent recorded in health record	13	8
Not documented	2	1
No response/not indicated	3	4

^a^ Respondents may indicate more than one provider who obtains IC from patients.

^b^ Institutions may deliver IC in more than one method.

Twenty-three of the respondents provided some degree of pre-test counseling and/or education to adult patients, seventeen respondents reported providing pre-test education to parents of pediatric patients and documenting it in the patient’s medical record, with only one respondent indicating that their institution did not record IC obtainment for pharmacogenomic testing (Table 6). Of the institutions that obtained IC from parents of pediatric patients, 12 (50%) also obtained assent from the pediatric patient as well. All respondents that provided pre-test education and obtained IC from both adult and parents of pediatric patients reported doing so in the same way for both populations. Time to obtain IC ranged from 0 to 60 min (including pre-test education and obtaining IC from patients), often taking less than 30 min for most sessions.

As it has the potential to impact considerations and practices regarding IC, data were collected regarding pharmacogenomic test details and methods utilized by the institutions. Thirty-five (81%) institutions reported testing for pharmacogenes with incidentalfindings. Of these, 19 (54%) responded that tests with incidental findings impacted their IC process to some degree, whereas 13 (37%) institutions indicated no impact to their IC process. Three (7%) institutions included pharmacogenes with incidental findings, but did not obtain IC from patients.

Twenty-one (49%) institutions utilized an external laboratory only, ten (23%) utilized internal laboratories only, and 12 (28%) utilized both external and internal laboratories. Recurring reasons cited for using an external testing site(s) include cost (relative to costs associated with internal testing), consideration of adherence to CPIC/FDA guidelines, integration options, accuracy, coverage, turnaround time, and ease of ordering. Of the 22 institutions that utilized an internal laboratory for pharmacogenomic testing to some extent, 18 (82%) employed genotyping technology, such as single nucleotide polymorphism (SNP) chip or array (Table 7).

**TABLE 7:** Pharmacogenomics testing methodologies utilized within internal laboratories (*n* = 22).

Methodology	n (%)^a^
SNP genotyping	18 (82)
Targeted sequencing	7 (32)
Whole exome sequencing	2 (9)
Whole genome sequencing	2 (9)
Real-time polymerase chain reaction	1 (5)
Unknown	1 (5)

^a^ Institutions may utilize multiple methodologies within their internal laboratories; percentage may exceed 100.

### 3.3 Reported caveats and barriers to informed consent

Survey respondents were asked if there were any additional details they wanted to share regarding patient education and IC for pharmacogenomic testing. Nine (21%) respondents stated that there are providers within their health system who may order testing without consulting the pharmacogenomics team, or that the pharmacogenomics team within their institution has varying degrees of involvement with different departments. Two respondents indicated that IC is not obtained for pharmacogenomic testing that is considered part of routine care (e.g., *DPYD* testing for oncology diagnoses).

**FIGURE 4:**
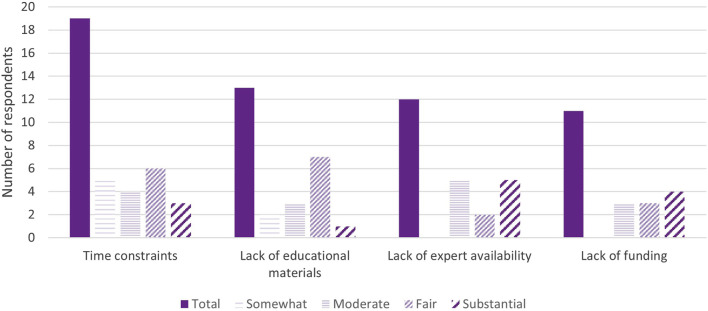
Major barriers for pharmacogenomic testing education/informed consent (*n* = 36). *Respondents may mark multiple options or not indicate the extent to which the barrier prevents them from obtaining IC.

Time was reported as a common barrier to providing further education and obtaining IC for pharmacogenomic testing; 19 (44%) respondents ranked it as at least a somewhat prohibitive factor. Lack of pharmacogenomics expert availability (28%) and funding (26%) were also cited as at least moderately deterring factors. Two respondents independently wrote that insurance and remote visits were moderately preventive factors for obtaining IC. Thirteen (31%) respondents did not indicate any factors preventing them from providing any/further possible patient education or IC for pharmacogenomic testing, two of which were institutions that did not obtain IC from patients (Figure 4).

### 3.4 Helpful resources for clinical implementation of pharmacogenomics

A majority of respondents (N = 43; 84%) indicated that one or more of the following resources would be helpful in further implementing pharmacogenomics at their institution: a consensus from a major pharmacogenomics organization with recommendations on pre-test education (84%), handouts on pharmacogenomic testing from a non-commercially affiliated/neutral 3^rd^ party resource (75%), and an “Ask the Experts” shared email consultation (51%). Over one-third (37%) of respondents reported wanting some form of additional support from their own institution (specifics not assessed). Two respondents also independently suggested fair use educational videos that may be shared with patients as part of pre-test counseling.

## 4 Discussion

Overall, the majority of respondents indicated that some form of IC was obtained from patients for pharmacogenomic testing with substantial overlap in the elements of IC; however, the responses demonstrated wide variability in the IC practices for pharmacogenomic testing at different clinical institutions. Our findings align with other reports for IC for pharmacogenomic testing (Haga and Mills, 2016) and, where overlapping, WES/WGS testing as well (Ayuso et al., 2013; Jamal et al., 2013; Fowler et al., 2017).

When IC was obtained, more than 90% of institutions discussed the purpose of testing and the general description of the test. Various other elements of IC were included in pre-test counseling to varying degrees (Tables 4, 5). The least common elements of IC incorporated into pre-test counseling were discussions on methodology, limitations of testing, accuracy of testing, and the implications of laboratory storage and potential usage of the sample for research and validation.

Compared to the previously identified eight elements commonly included in disease-based genetic testing, survey respondents reported covering many of the same elements with a few notable differences (Table 5). First, the description of the condition being tested does not apply in pharmacogenomic testing and/or is somewhat assumed – medicine response is being tested either pre-emptively or reactively, and as such was not queried. Additionally, the medical risks and benefits of undergoing testing were not specifically queried as it is assumed to be similar to other forms of laboratory testing. Rather, the specimen collection for pharmacogenomic testing is typically acquired with a buccal sample or in some cases a blood draw, both of which are generally covered under the institution’s consent to treat. The cognitive risks and benefits are possibly more impactful in the context of gaining information on potential medicine response with the identified risks of incidental findings and/or biological relatedness in the setting of testing multiple individuals.

Discussion of methodology of testing is often excluded in pre-test counseling since it may be considered by providers to be irrelevant or difficult information for patients to understand (Luksic et al., 2020). The methodology and accuracy of genetic testing is important for the provider ordering testing to understand in order to make the appropriate choice of assay for the patient and recognize the limitations of that assay. This is of more concern in molecular diagnostics where a formal diagnosis is being obtained, as different types of genetic variants may only be detected through certain methodologies, such as Sanger sequencing for sequence variants (Pareek et al., 2011) versus multiplex ligation-dependent probe analysis for copy number variants (i.e., deletions and duplications) (Stuppia et al., 2012). Further, a person’s genotype for a particular pharmacogene is not the sole contributor to their response to an associated drug; it is well understood that pharmacogenomic test results should be only one of many factors (e.g., organ health, environmental exposures, concurrent medications, etc.) considered for medication selection or dosing decisions (Almazroo et al., 2017). In addition, methodology or accuracy may not be widely communicated to patients in pre-test pharmacogenomic counseling because non-genetics providers may be less familiar with the nuances of different genetic testing methodologies (Miller et al., 2014; Conway et al., 2019), and therefore, feel less comfortable in discussing this test detail.

Another important consideration that may impact patient education and IC practices is the patient population with respect to ancestry and varying benefits of testing. In particular, the under-representation of diverse populations in pharmacogenomic testing/research may limit the utility of testing for these groups (National Academies of Sciences, 2018; Williams et al., 2018; Zhang and Finkelstein, 2019). Currently, most pharmacogenomic testing is performed through genotyping methodologies and thus, will only detect select variants (Caspar et al., 2021), which are likely biased for populations of European ancestry. Indeed, as evidenced by our findings, many clinical institutions using an internal laboratory for pharmacogenomic testing are primarily using SNP chip and/or array technology (Table 6), which is also commonly utilized by commercial pharmacogenomics laboratories (Lemieux Perreault et al., 2018). For patients from these less-studied populations, a negative finding (or normal) may actually be an uninformative negative result. This inequity in pharmacogenomic testing demonstrates the importance of discussing methodology with patients to the extent that they understand that the reported genotype may not accurately represent their phenotypic metabolizer status.

Potential implications for relatives demonstrated the widest disparity between IC practices of adult patients and parents of pediatric patients, with less than half of the surveyed institutions including this element for adult patients. Additionally, significant incidental findings are rarely detected in pharmacogenomic testing (Westbrook et al., 2013; Haga, 2021). Thus, practitioners who may rarely encounter these incidental findings likely would not counsel patients on this possibility due to limited understanding of their implications (Coleman et al., 2023).

Obtaining IC for pharmacogenomic testing poses several logistic and unique challenges, some of which may support an abbreviated IC process (Ando and Terada, 2023). While there is undeniably value in sharing the benefits, limitations, and alternatives to testing, genetic testing in general can be inherently more difficult to explain to patients compared to other medical interventions due to the complexity of genetics concepts (Christensen et al., 2010). Among the challenges to obtaining IC are health literacy, time, and provider knowledge and skill. Health literacy is an important issue to consider and can at times be difficult to reconcile with providing sufficient information to the patient such that they can act autonomously in the best interest of their health. It has been reported that patient comprehension and recall are limited even following receipt of additional educational materials about pharmacogenomics during the course of their care (Haga et al., 2014; Olson et al., 2017; Asiedu et al., 2020). Given that genetic testing is complex with several different issues, this can further extend the average consultation time with a patient (Bester et al., 2016). Within healthcare there is pressure to balance the amount of information provided to patients within the already limited time providers have available to provide additional services. This is the case for pharmacogenomics where other barriers already stand in the way of widespread implementation (Ando and Terada, 2023). Thus, more clear guidelines regarding what to include and the degree of IC needed for pharmacogenomic testing becomes even more relevant given the range of different types of genetic tests, clinical settings, access to genetic specialists, and various other implementation factors (Haga et al., 2013; Rego et al., 2019).

The results of our survey suggest pharmacists play a key role in pharmacogenomics clinics and testing workflow across institutions as they made up the majority of our survey respondents. This was expected given the patterns reported in previous literature (Haga et al., 2017), and is in-line with professional statements on the roles of pharmacists in clinical pharmacogenomics (Hicks et al., 2019; Haidar et al., 2021). It is worth noting that neither pharmacists nor genetic counselors are currently considered to have “provider” status per the U.S. Centers for Medicare and Medicaid Services (CMS). As such, reimbursement for pharmacogenomics services necessitates, and often further benefits from, involvement of a billable provider (Dunnenberger et al., 2016). Recent literature has demonstrated the benefit of a collaborative model between pharmacists and GCs in a pharmacogenomics clinic (Mills and Haga, 2013; Zierhut et al., 2017; Gammal and Fieg, 2022). Pharmacists possess expertise in the pharmacokinetics and pharmacodynamics of medication use and are able to provide treatment recommendations based on pharmacogenomic test results with proper training. GCs are trained to communicate the details, facilitate the shared decision-making process for, and interpret the results of genetic testing (Accreditation Council for Genetic Counseling, 2023) and they have in-depth training and expertise on molecular diagnostic testing which can impact interpretation. Together, these providers can provide a more comprehensive approach to pre-test counseling for clinical pharmacogenomic testing.

Despite this ideal collaboration, several logistic difficulties prevent this partnership, including the limited number of currently practicing GCs. As of March 2023, there were approximately 6,641 GCs in the United States; however, this number is expected to grow to over 10,000 by the year 2030 (NSGC, 2023). Despite this high rate of growth, there is a high demand for GCs across all specialties within the field, limiting the number of GCs who can be involved in pre-test pharmacogenomic counseling. While a majority of GCs report receiving some form of pharmacogenomics education in their graduate training, many report that they do not feel well enough informed about pharmacogenomic testing, some citing limited knowledge and discomfort discussing details of medication therapy (Haga et al., 2012; Loudon et al., 2021).

One component to consider with genetic testing, including pharmacogenomic testing, when obtaining IC is “genetic exceptionalism”, where all genetic information must be treated differently from other forms of medical information, especially in cases of predictive testing (Buchanan et al., 2002; Mannette, 2021). However, with the increasing use, familiarity, and acceptability of genetic testing in healthcare, the utility and practicality of genetic exceptionalism in medicine is being debated (Witt and Witt, 2016; Garrison et al., 2019; Terry, 2020), especially in the case of pharmacogenomic testing (Buchanan et al., 2002; Relling et al., 2010). The potential for incidental findings presents a stronger argument in favor of providing some degree of patient education or IC, which was reflected in over 73% of respondents having indicated that including incidental findings impacted their IC process (Table 5). While the focus of testing is medicine response, notably, some of the queried genes have the potential to inform on either health conditions and/or carrier status and may warrant additional patient discussion as to avoid unexpected findings after testing. Of note, the American College of Medical Genetics (ACMG) advises upon reporting of secondary findings in the context of WES; however, the only pharmacogenes noted in the 2021 ACMG policy statement that are relevant to pharmacogenomics are *CACNA1S* and *RYR1* (Miller et al., 2021). Overall, our data suggest that clinical institutions tend to focus on the general concepts of what pharmacogenomic testing is and what it entails for the patient. Other elements of IC were included to varying degrees, underscoring the need and potential value for further investigation regarding whether and what elements of IC should be offered to patients tested in the clinical (i.e., non-research) setting.

There are some limitations that survey-based research imposes on the collection, analysis, and generalization of data. In order to make the survey less time-consuming for prospective respondents, the survey questions did not delve into too much detail of particular nuances of each institution’s IC practices. The sample size was relatively small and was solicited from groups of individuals more likely to be involved in pharmacogenomics within their institution. There are also inherent biases within self-reported surveys (Althubaiti, 2016), such as recall bias. Because not all of the respondents were directly involved in the patient education and IC process, there may also be some inaccuracies to some of the responses. We attempted to limit recall bias by allowing respondents the opportunity to pause the survey and return to it, should they have felt the need to refresh themselves on their institution’s IC process and discuss with colleagues. Further, although our targeted survey population was anticipated to have a higher likelihood of exposure and utilization of pharmacogenomic testing, it is possible that important stakeholders were missed.

Future directions for this topic include further work towards developing consensus recommendations for IC for pharmacogenomic testing and the creation of handouts and/or video resources created and made available from a non-commercial, neutral third party without actual or perceived conflicts of interest for patients and providers alike. Further research in this area may be valuable to determine what providers would prefer to be included in these educational materials and subsequent effectiveness on patient understanding. Educational materials for providers may also facilitate further clinical implementation of pharmacogenomic testing to increase providers’ level of comfort with ordering and discussing testing with patients.

In conclusion, as the demand for pharmacogenomic testing grows, so too does the need for medical professionals who are properly prepared to discuss the pertinent details of testing so that patients may understand what this testing can and cannot provide. The responses to our survey demonstrate the wide variability across and within clinical institutions with respect to patient education and IC for pharmacogenomic testing, which may be remedied by the establishment of consensus recommendations for IC practices. This study has demonstrated important insights into the current landscape of pharmacogenomics practices and how clinical implementation may be improved upon in the future.

## Data Availability

The datasets presented in this article are not readily available because The REDCap raw data [in cases] identify institutions and individuals who voluntarily provided their contact information. The invitation expressly states that the data will not be reported in an identifiable manner. Email EL for further questions. Requests to access the datasets should be directed to EL, ejwleary@gmail.com.
