# Supplementary material for: Assessment of the current status of real-world pharmacogenomic testing: informed consent, patient education, and related practices
Source: Front Pharmacol. 2024 Feb 8;15:1355412. doi: 10.3389/fphar.2024.1355412 (PMC10895424; doi:10.3389/fphar.2024.1355412)
Supplement: Supplementary file 1 [file DataSheet2.pdf]

**Multi-Institutional Survey on Informed Consent Practices in Clinical Germline Pharmacogenetic Testing**
**v10/26/22**

# Preamble:

**Marshfield Clinic Research Institute – Center for Precision Medicine Research Participant Information and Consent Form**

- **Study title**: Multi-Institutional Survey on Informed Consent Practices in Clinical Germline Pharmacogenetic Testing

- **Study team** (alphabetical order): Anna Cisler, MS, CGC; Susanne Haga, PhD; Cyrine Haidar, PharmD; April Hall, PhD, MS, CGC; Scott Hebbring, PhD; Emili Leary, PharmD; Lucas Pereira, BA; Sanjay Shukla, PhD

**Description of Research**
You are invited to participate in a research study about the patient education/informed consent practices of germline pharmacogenetic (PGx) testing in a clinical setting (note, germline = not tumor tissue testing). Please ONLY take this survey if your institution offers clinical pharmacogenetic testing to patients. This survey is open to all organizations and individuals who are actively involved in clinical germline pharmacogenetic testing, thus if you have colleagues or collaborators that may be willing to provide responses please feel free to share this invitation with them. **Please only submit one survey per person.**

This study aims to elicit current practices regarding patient education, informed consent and/or informed assent processes across different clinical institutions and to analyze the trends. While previous research has investigated patient education/informed consent in research and laboratory settings, there is currently no uniform practice about whether and how to obtain and document patient education and/or informed consent/assent prior to PGx testing. This survey is intended to elucidate the current status of these practices for PGx testing, and work towards addressing the need for establishing standards for patient education and informed consent/assent regarding clinical PGx testing.

**What does your participation involve?**
If you choose to participate in this research, you will be asked to complete an online survey. This survey has at minimum 3 questions and at most >40 questions; estimated completion time ranges from 5-20 minutes depending upon your answers. Your participation is voluntary, you may skip any questions that you do not want to answer, and you may stop participating at any time by simply closing your web browser. Should you choose to participate, you will be asked questions pertaining to whether your institution provides patient education/informed consent for clinical PGx testing and (if applicable) what current patient education/informed consent practices are done prior to PGx testing.

**Are there any risks to you, your patients, or your institution?**
There are no anticipated risks to partaking in this study. Please do not share any protected health information (PHI) such as date of birth, medical history number, etc. about individuals you serve. Should you feel uncomfortable in taking the survey, you may skip any questions that cause discomfort.
Your responses will be kept confidential and the information you provide will not be published in a way that would allow for you to be identified. Participating institutions may be identified in the published peer-reviewed manuscript(s) resulting from this research. Quotes/open-ended responses may be paraphrased and data may be aggregated for publication. Attribution of quotes to individuals from

institutions may be anonymized if desired. If you wish to withdraw your consent (which can be done up to the point at which the manuscript has been submitted for publication) please contact Emili Leary, PharmD at 715-221-7090 or email at Leary.Emili@MarshfieldClinic.org.

**Whom can you contact with questions or concerns?**
You may ask any questions about this research study at any time. If you have questions about the research, please contact the Lead Co-Investigators:

Emili Leary, PharmD
Research, Clinical, MTM and Outpatient Pharmacist
Marshfield Clinic Research Institute - Center for Precision Medicine Research
leary.emili@marshfieldclinic.org

Lucas Pereira, BA
Genetic Counseling Intern 2023
University of Wisconsin - School of Medicine and Public Health
pereira.lucas@marshfieldclinic.org

If you would like a copy of this information for your records, select the PDF attachment below.

**By clicking next, you are providing consent to participate. You may stop and submit your responses at any time without penalty.**

**Multi-Institutional Survey on Informed Consent Practices in Clinical Germline Pharmacogenetic Testing**
**v10/26/22**

## Questionnaire Content

1. **Question 1**: Please provide the name and location of the institution you practice at:
    a. Text box

2. **Question 2** (Branch Logic for Question groupings 4, 11, 18): Which of the following patient populations are offered pharmacogenetic (PGx) testing at your institution? (Radio Buttons)
    a. No clinical pharmacogenetic testing is offered at all at my institution (Branch Logic: skips to question 43 [*may we contact you if questions*] and then closes survey)
    b. Adults (≥18 years; Branch 1)
    c. Pediatrics (<18 years; Branch 2)
    d. **Both** (includes Branch 1 and Branch 2)
    e. Other (text box; expands Branch 1 and Branch 2)

3. **Question 3 (**Branch Logic for Questions 4, 11, 18**):** Does your institution have specific guidelines/practices about patient education, informed consent and/or informed assent prior to PGx testing for any clinically tested population? (Radio buttons)
    a. No – my institution does NOT do any patient education, informed consent, and/or informed assent prior to PGx testing (skips questions 4-26)
    **b.** I do not know/Additional comment (text box; expand to following questions when selected, and selection guided by answer to Question 2)
    c. Yes – our institution does some degree of patient education, informed consent, and or/informed assent prior to PGx testing (expand to following questions when selected, and selection guided by answer to Question 2)

4. **Question 4** (Branch Logic: Branch 1: *Adult or "**Both**" selected*): Is pre-test education and/or consenting provided to adult patients and/or their legal guardian at your institution? (Radio Buttons for Y/N; Branch Logic to expand or hide further selections when a box is checked)
    a. **No** pre-test patient education/consenting done for adults and/or their legal guardian (Branch Logic: Skips/hides following questions 5-10)
    b. **I do not know** if patient education/consenting is done for adults and/or their legal guardian (Branch Logic: Skips/hides following questions 5-10)
    c. **Yes**, pre-test patient education/consenting is done for adults and/or their legal guardian (Branch Logic: Expand to make questions 5-10 available)
        a. **Question 5:** What pre-test education/consenting elements are included? (Select all that apply; Branch Logic to expand further selections when a box is checked)
            i. Benefits of testing
                1. Assist with medication selection/avoidance
                2. Lifetime use of results
            ii. Risks/Considerations of testing
                1. Patient Protections (e.g., GINA, HIPAA, State Laws, etc.)
                2. Secondary/Incidental Findings (e.g., genes DPYD, F5, X-linked genes, etc.)
                3. Biological relatedness and/or implications of results for biologically-related individuals
                4. When genetic counseling may be recommended
                5. Laboratory storage and use of sample for validation, research, etc.

iii. Test education/use of results
    1. General description of the test
    2. Purpose of testing
    3. Accuracy of testing
    4. Actionability of results
    5. Limitations to testing – general limitations
    6. Limitations to testing – methodology limitations
    7. Cost of testing
    8. Interpretation of Results

iv. I know we do provide education/consent, but I do not know what is covered/Explain (text box)

v. If there are additional comments/components/caveats included in the pre-test education/counseling performed at your institution, please list them here (e.g., only do x, y, z in certain settings…etc.). (Text/Comment Box)

b. **Question 6:** How is this education/consenting done? (Select all that apply)
    i. In person
    ii. By phone
    iii. By Telehealth Video
    iv. Written communication
    v. I do not know/Other (please explain) (text box)

c. **Question 7:** Is this pre-test education/consenting recorded in the patient's medical record? (Radio buttons)
    i. No (Branch logic to skip to question 9)
    ii. I do not know/Other (text box)
    iii. Yes (Branch Logic – expand to next question)
        1. **Question 8**: Is consent for testing obtained from the patient and/or their legal guardian? (Radio buttons)
            a. Yes – signed consent for testing is obtained and is documented in the medical record
            b. Yes – signed consent for testing is obtained **but not** documented in the medical record
            c. Yes – verbal consent for testing is obtained and is documented in the medical record
            d. Yes – verbal consent for testing is obtained **but not** documented in the medical record
            e. No – no consent for testing is obtained from the patient, only education is provided
            f. Other (text box)

d. **Question 9:** Who conducts this pre-test education/consenting to the patient/legal guardian (please list all that apply and please indicate who is the most frequent individual to do this – e.g., medical assistant, nurse, clinician, pharmacist, genetic counselor, etc.)?
    i. [Text/Comment Box]

e. **Question 10:** How much time is typically spent by the provider who provides this education/consent information? (Select all that apply; this question terminates/is the last question in Branch 1 only selection)

i. < 15 minutes
ii. 15-30 minutes
iii. 31-45 minutes
iv. 46-60 minutes
v. > 60 minutes
vi. I do not know/Other (text box)

5. **Question 11**: (*Branch 2: Pediatric or "**Both**" selected*): Is pre-test education and/or consenting provided to the parents/legal guardians of the pediatric patients at your institution? (Radio Buttons for Y/N; Branch Logic to expand or hide further selections when a box is checked)
    a. **No** pre-test patient education/consenting is provided to the parent(s)/legal guardian(s) of the pediatric patients (Branch Logic: Skips /hides questions 12-17)
    b. **I do not know** if patient education/consenting is provided to the parent(s)/legal guardian(s) of the pediatric patients (Branch Logic: Skips /hides questions 12-17)
    c. **Yes**, pre-test patient education/consenting is provided to the parent(s)/legal guardian(s) of the pediatric patients (Branch Logic to expand to following questions when selected)
        a. **Question 12:** What pre-test education/consenting elements are included? (Select all that apply and text box; Branch Logic to expand further selections when a box is checked)
            i. Benefits of testing
                1. Assist with medication selection/avoidance
                2. Lifetime use of results
            ii. Risks/Considerations of testing
                1. Patient Protections (e.g., GINA, HIPAA, State Laws, etc.)
                2. Secondary/Incidental Findings (e.g., genes DPYD, F5, X-linked genes, etc.)
                3. Biological relatedness and/or implications of results for biologically related individuals
                4. When genetic counseling may be recommended
                5. Laboratory storage and use of sample for validation, research, etc.
            iii. Test Education/Use of Results
                1. General description of the test
                2. Purpose of testing
                3. Accuracy of testing
                4. Actionability of results
                5. Limitations to testing – general limitations
                6. Limitations to testing – methodology limitations
                7. Cost of testing
                8. Interpretation of Results
            iv. I know we do provide education/consent, but I do not know what is covered/Explain (text box)
            v. If there are additional comments/components/caveats included in the pre-test education/counseling performed at your institution, please list them here (e.g., only do x, y, z in certain settings…etc.). (Text/Comment Box)
        b. **Question 13:** How is this education/consenting done? (Select all that apply)

   i. In person
   ii. By phone
   iii. By Telehealth Video
   iv. Written communication
   v. I do not know/Other (please explain) (text box)

c. **Question 14:** Is this pre-test education/consenting recorded in the patient's medical record? (Radio buttons)
   i. No
   ii. I do not know/Other (text box)
   iii. Yes (Branch Logic – expand to next question)
      1. **Question 15**: Is consent for testing obtained from the patient's parent(s)/legal guardian(s)? (Radio buttons)
         a. Yes – signed consent for testing is obtained and is documented in the medical record
         b. Yes – signed consent for testing is obtained **but not** documented in the medical record
         c. Yes – verbal consent for testing is obtained and is documented in the medical record
         d. Yes – verbal consent for testing is obtained **but not** documented in the medical record
         e. No – no consent for testing is obtained from the patient's parent(s)/legal guardian(s), only education is provided
         f. I do not know/Other (explain) (text box)

d. **Question 16:** Who conducts this pre-test education/consenting to the patient/caregiver (please list all that apply and please indicate who is the most frequent individual to do this – e.g., medical assistant, nurse, clinician, pharmacist, genetic counselor, etc.?
   i. [Text/Comment Box]

e. **Question 17:** How much time is typically spent by the provider who provides this education/consent information? (Select all that apply)
   i. < 15 minutes
   ii. 15-30 minutes
   iii. 31-45 minutes
   iv. 46-60 minutes
   v. > 60 minutes
   vi. I do not know/Explain (text box)

6. **Question 18**: (*Branch 2: Pediatric or "**Both**"*): Which of the following elements of pre-test education and/or assent is provided to the pediatric patients themselves as part of the assent process? (Radio buttons for Y/N; survey logic to expand or hide further selections when a box is checked)
   a. **No pre-test pediatric patient education/assent is done at all** (Branch Logic: Skips/hides questions 19-25)
   b. **I do not know** if pediatric patient education/assent is done (Branch Logic: Skips/hides questions 19-25)

c. **Yes**, assent is provided and contains the same elements as provided to the pediatric patient's parents/legal guardians. (Branch Logic: Skips/hides following questions)

d. **Yes**, assent is provided and contains different elements than provided to the pediatric patient's parents/legal guardians. (Select all that apply and Branch Logic to expand questions as below)

   i. **Question 19**: When utilizing PGx testing for a pediatric patient, what is the minimum age (in years) in which the patient is involved for attempting obtaining assent?
      1. (sliding scale up to and including 17 years)
      2. I do not know/Other (text box)

   ii. **Question 20:** What pre-test education/assenting elements are included?
      1. Benefits of testing
         a. Assist with medication selection/avoidance
         b. Lifetime use of results
      2. Risks/Considerations of testing
         a. Patient Protections (e.g., GINA, HIPAA, State Laws, etc.)
         b. Secondary/Incidental Findings (e.g., genes DPYD, F5, X-linked genes, etc.)
         c. Biological relatedness and/or implications of results for biologically related individuals
         d. When genetic counseling may be recommended
         e. Laboratory storage and use of sample for validation, research, etc.
      3. Test Education/Use of Results
         a. General description of the test
         b. Purpose of testing
         c. Accuracy of testing
         d. Actionability of results
         e. Limitations to testing – general limitations
         f. Limitations to testing – methodology limitations
         g. Cost of testing
         h. Interpretation of Results
      4. I know we do provide pediatric assent, but I do not know what is covered/Explain (text box)
      5. If there are additional comments/components/caveats included in the pre-test education/assenting performed at your institution, please list them here (e.g., only do x, y, z in certain settings…etc.). (Text/Comment Box)

   iii. **Question 21:** How is this education/assenting done?
      1. In person
      2. By phone
      3. By Telehealth Video
      4. Written communication
      5. I do not know/Other (please explain) (text box)

   iv. **Question 22:** Is this pre-test education/assenting recorded in the patient's medical record? (Radio buttons)
      1. No

2. I do not know/Other (text box)
3. Yes (Branch Logic – expand to next question)
   a. **Question 23**: Is assent for testing obtained from the patient? (Radio buttons)
      i. Yes – signed assent for testing is obtained and is documented in the medical record
      ii. Yes – signed assent for testing is obtained **but not** documented in the medical record
      iii. Yes – verbal assent for testing is obtained and is documented in the medical record
      iv. Yes – verbal assent for testing is obtained **but not** documented in the medical record
      v. No – no assent for testing is obtained from the patient, only education is provided
      vi. I do not know/Other (text box)

v. **Question 24:** Who conducts this pre-test education/assenting to the patient (please list all that apply and please indicate who is the most frequent individual to do this – e.g., medical assistant, nurse, clinician, pharmacist, genetic counselor, etc.?
   1. [Text/Comment Box]

vi. **Question 25:** How much time is typically spent by the provider who provides this education/assent information? (select all that apply; this question terminates/is the last question in Branch 2 or "**Both**" selections)
   1. < 15 minutes
   2. 15-30 minutes
   3. 31-45 minutes
   4. 46-60 minutes
   5. > 60 minutes
   6. I do not know/other (text box)

vii. **Question 26**: At age 18, is the patient contacted to consent? (Radio buttons)
   1. Yes
   2. No
   3. I do not know/Other (text box)

7. **Question 27**: Does your institution perform in-house PGx testing or does it use an external laboratory for clinical pharmacogenomics testing of patients? (Branch/Logic Point; Select all that apply)
   a. I do not know/other (explain) (text box; branch logic to skip questions 28-31)
   b. In-house (Branch 1)
      i. **Question 28:** What is your methodology? (Select all that apply plus text box)
         1. Genotyping (SNP chip/array technology)
         2. Targeted Sequencing
         3. Whole Exome Sequencing
         4. Whole Genome Sequencing
         5. Other (describe)
         6. I don't know/Unknown

c. External Company (Branch 2)
   i. **Question 29:** Which external laboratory/laboratories does your institution use? (text box)
   ii. **Question 30**: What influenced your institution's selection of laboratory/laboratories to use? (text box)

d. **Both**/Mix/Dependent on gene/drug (**Both**: includes both Branches 1 and 2 plus below question)
   i. **Question 31:** If you use **both**, what determines the decision? (text box; This question terminates/is the last question in this branch)

8. **Question 32**: Does your institution test for, or use an external company that tests for any pharmacogenes with secondary/incidental findings (e.g., *ApoE, BCHE, CACNA1S, DPYD, F2, F5, G6PD, GBA, GGCX, HTR2C, MTHFR, MT-RNR1, NAGS, POLG, RYR1, SCN1A, SLCO1B1* and *SLCO1B3, UGT1A1*, X-linked genes, etc.)? (Select all that apply, but if possible keep selection of Yes and No mutually exclusive.)
   *a.* No (branch logic to skip question 33)
   *b.* I do not know/Other/Please explain (text box; branch logic to skip question 33)
   *c.* Yes (expand to next question)
      *i.* **Question 33**: (New Branch/expansion) By including testing for genes with secondary findings, did that impact what elements you include in your patient education/consenting/assenting? (Select all that apply and text box)
         *1)* Yes
         *2)* No
         *3)* Somewhat
         *4)* I'm not sure
         *5)* Explain (Text Box)

9. **Question 34**: To what extent do the following factors prevent you from providing any/further possible patient education/consenting on PGx? (Select all that apply and Likert scale 1-5 for each selected item: 1=Not at all, 2=Somewhat, 3=A Moderate Amount, 4=A Fair Amount, 5=Substantially)
   a. PGx Expert availability
   b. Time
   c. Funding
   d. Lack of educational materials to share with patients
   e. None/NA
   f. Other(s); please assign Likert Scale rating to rate each additional factor that may prevent you from providing further education/consultation (text box)

10. **Question 35**: Please feel free to share any additional details/caveats about your practice site and patient education/consenting for PGx (e.g., only do reactive testing for a given indication, only Psychiatry department uses testing, etc.) (text box)

**Multi-Institutional Survey on Informed Consent Practices in Clinical Germline Pharmacogenetic Testing**
**v10/26/22**

## Demographic Data

11. **Question 36**: Over the last year, how many pharmacogenetic (PGx) tests were ordered per month (on average) at your institution for adult patients?
    a. 0 (None)
    b. 1-2
    c. 3-5
    d. 6-10
    e. 11-20
    f. >20
    g. Unknown

12. **Question 37**: Over the last year, how many pharmacogenetic (PGx) tests were ordered per month (on average) at your institution for pediatric patients?
    a. 0 (None)
    b. 1-2
    c. 3-5
    d. 6-10
    e. 11-20
    f. >20
    g. Unknown

13. **Question 38**: In which of the following settings is pharmacogenetic (PGx) testing offered at your institution? (Select all that apply)
    a. Ambulatory/Outpatient Clinic Care Setting
    b. Hospital/Inpatient Care Setting
    c. Industry/PGx Testing Company
    d. I don't know/Other (explain) (text box)

14. **Question 39:** What is your position title/Specialty (e.g., Primary Care Clinician, Oncology Genetic Counselor, Infectious Disease Pharmacist, etc.) at your institution?
    a. Position Title/Specialty: (text box)

15. **Question 40**: What is your educational degree (e.g., MD, MS – Genetic Counseling, PharmD, etc.) at your institution? (Select all that apply; Branch Logic to expand to educational degree)
    b. Pharmacist
        i. PharmD
        ii. RPh
    c. Physician
        i. MD
        ii. DO
        iii. MBBS
        iv. PhD (e.g., Psychologist)
        v. Additional board certification (text box)
    d. Nurse Practitioner
        a. What is your primary specialty? (text box)
    e. Physician Assistant

        i. What is your primary specialty? (text box)

   f. Genetic Counselor

        ii. What is your primary specialty? (select all that apply)

            1. Cardiology
            2. Neurology
            3. Oncology
            4. Prenatal Care
            5. Pediatrics
            6. Psychiatry
            7. Other (text box)

   g. Other (text box)

16. **Question 41:** How long have you been working as a professional in your current field of practice? (radio buttons)
    a. <1 year
    b. 1-2 years
    c. 3-5 years
    d. 6-10 years
    e. >10 years
    f. Other (explain) (text box)

17. **Question 42**: What is your education training in PGx? (can select multiple boxes, text box)
    a. Master's degree in PGx
    b. Part of formal educational training program within primary degree
    c. Took PGx Certificate Course
    d. PGY2 in PGx
    e. Continuing education
    f. On the job training (e.g. informal education provided by colleagues or your institution)
    g. No education in pharmacogenetics
    h. Other (text box)

18. **Question 43**: If we have questions on your responses may we contact you for clarification?
    a. Yes → text box for name and email address
    b. No (radio button)

**Multi-Institutional Survey on Informed Consent Practices in Clinical Germline Pharmacogenetic Testing**
**v10/26/22**

## Additional Optional Questions

Optional extension – "Do you want to answer a couple more questions?" (Yes/No radio buttons; Yes – expand to include last 2 questions, No – Take to submission page)

19. **[Optional] Question 44**: If available, which of the following resources would you like to see become available to use to provide PGx pre-test education to patients? (Select all that apply)
    a. EXTERNAL: Consensus from a major PGx organization with consensus recommendations on pre-test education and content
    b. EXTERNAL: Handouts on PGx testing from a non-commercially affiliated/neutral 3rd party resource
    c. EXTERNAL: An "Ask The PGx Experts" shared email hosted by an external expert group for questions
    d. INTERNAL: Additional support from/at my institution
    e. Other(s) (text box)

20. **[Optional] Question 45**: If allowed/appropriate, please share any documents related to your (personal or institutional) PGx educational materials. *You may also email these to Leary.Emili@MarshfieldClinic.org at a later date and indicate it as part of your survey response*. (upload option)

**Multi-Institutional Survey on Informed Consent Practices in Clinical Germline Pharmacogenetic Testing**
**v10/26/22**

Message following survey submission:

Thank you for participating in this survey, we hope to publish our findings in 2023!

For any questions/concerns please contact:

Emili Leary, PharmD
Research, Clinical, MTM and Outpatient Pharmacist
Marshfield Clinic Research Institute - Center for Precision Medicine Research
leary.emili@marshfieldclinic.org

Color Key:

**Purple** – identifies the question

**Blue** – identifies answer/response formatting and communicates information to the REDCap programmer

**Red** – identifies Branch Logic; also used to denote termination of a branch.

**Orange** – denotes upcoming branch expansion/extension for additional questions

**Green**/**Pink** – denote and help track branching logic paths between two options
