# Supplementary material for: Assessment of the current status of real-world pharmacogenomic testing: informed consent, patient education, and related practices
Source: Front Pharmacol. 2024 Feb 8;15:1355412. doi: 10.3389/fphar.2024.1355412 (PMC10895424; doi:10.3389/fphar.2024.1355412)
Supplement: Supplementary file 2 [file DataSheet1.docx]

# Supplementary Material

**Figure 1**. Survey responses by institution.

**Figure 2**. States within the United States with a clinical pharmacogenomic respondent institution in purple.


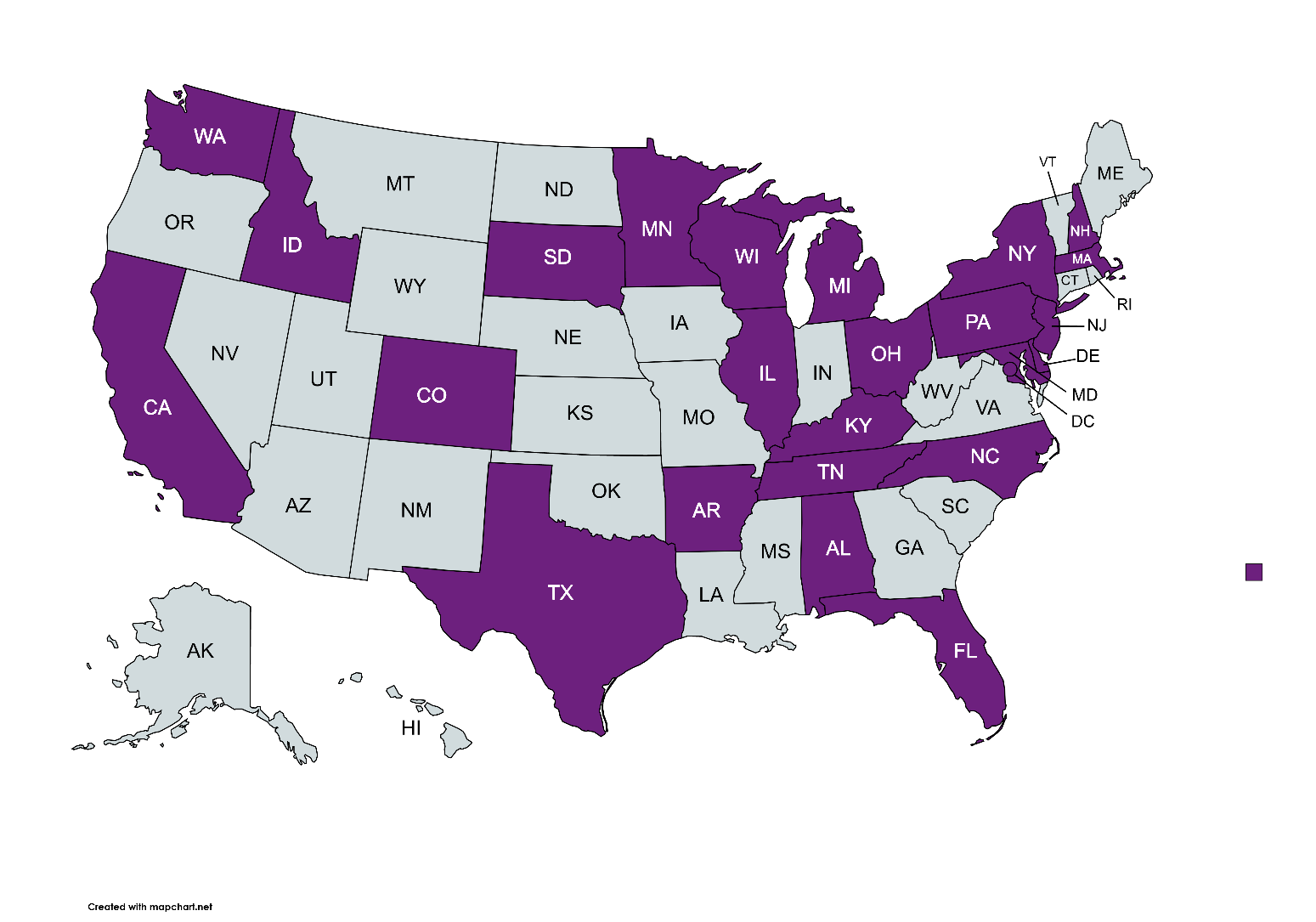


**Figure 3**. Institutions’ IC Practices by Age Demographic (n = 43).


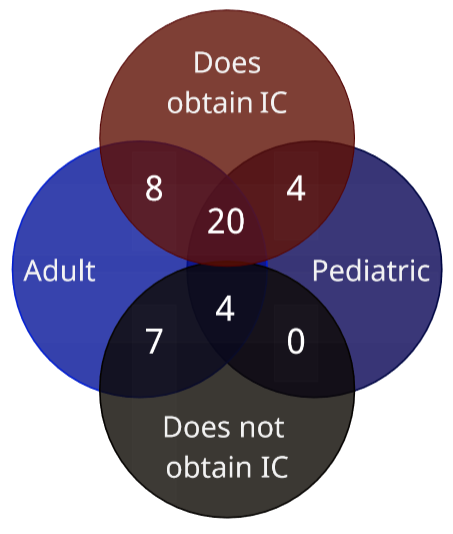


**Figure 4**. Major barriers for pharmacogenomic testing education/informed consent (n = 36).

**Respondents may mark multiple options or not indicate the extent to which the barrier prevents them from obtaining IC*
